# Optimisation of the Thin-Walled Composite Structures in Terms of Critical Buckling Force

**DOI:** 10.3390/ma13173881

**Published:** 2020-09-02

**Authors:** Karol Szklarek, Jakub Gajewski

**Affiliations:** Department of Machine Design and Mechatronics, Faculty of Mechanical Engineering, Lublin University of Technology, Nadbystrzycka 36, 20-618 Lublin, Poland; j.gajewski@pollub.pl

**Keywords:** laminates, ply orientation, optimisation, FEM, ANN, artificial neural network, parametric studies, thin-walled, composites

## Abstract

The paper presents the optimisation of thin-walled composite structures on a representative sample of a thin-walled column made of carbon laminate with a channel section-type profile. The optimisation consisted of determining the configuration of laminate layers for which the tested structure has the greatest resistance to the loss of stability. The optimisation of the layer configuration was performed using two methods. The first method, divided into two stages to reduce the time, was to determine the optimum arrangement angle in each laminate layer using finite element methods (FEM). The second method employed artificial neural networks for predicting critical buckling force values and the creation of an optimisation tool. Artificial neural networks were combined into groups of networks, thereby improving the quality of the obtained results and simplifying the obtained neural networks. The results from computations were verified against the results obtained from the experiment. The optimisation was performed using ABAQUS^®^ and STATISTICA^®^ software.

## 1. Introduction

Today, composite materials tend to replace elements formerly made of aluminium or steel. One of the frequently used composite materials is laminate. The mechanical properties of the manufactured laminate can be modified by implementing various types of laminae in the form of tapes or fabrics. Additionally, a single layer of laminate may be arranged at different angles, and the obtained configurations display substantial differences in strength properties [1]. Therefore, it is important to select such a laminate that fulfils all the design and safety requirements. For the manufacturing simplicity, the most frequent angle configurations are 0°, 90°, 45°, ±45°. The angle configurations concern tapes and fabrics used to produce laminates. Lately, the usage of different angles, such as ±30°, ±60° [2,3,4,5,6,7,8,9,10,11], has been debated. In the case of tubes and pressure vessels, the angle of fibre location (depending on the target strength properties) is determined to the nearest 1°. Due to the complexity of the problem, the optimisation of composite structures is more difficult and poses an interesting problem for the researchers [12,13,14]. The problem of stability in carbon composite (CFRP) thin-walled profiles with open cross-sections has been widely studied [15,16,17,18,19,20,21,22]. The structure loses its stability when the load reaches or exceeds a certain force value called buckling critical force or buckling load. After reaching this value, the structure is most often damaged and may be considered unfit for further operation. However, there are many structures, a large part of which are made of composites, that retain stability after exceeding the critical force values; however, their stiffness changes. An example of such a structure can be a truss bridge, which changes its stiffness due to unforeseen circumstances but does not lose its stability. The behaviour of these structures after exceeding the critical force is studied with nonlinear post-buckling analyses. Another important aspect is the loss of stability, both local and global. The local loss of stability occurs when only a part of the structure loses its stability, while the global loss concerns the loss of stability in the entire structure. With respect to the truss bridge, the local loss of stability will occur when one span is bent, while the global loss will occur when the bridge collapses or tilts. There is also a possibility of cooperation of local and global modes of buckling, which may result in high instability after buckling of the structure and sensitivity to errors. Therefore, at the design and optimisation stage, both global and local loss of stability must be accounted for. The manufacturing process can lead to design errors that may reduce the effects of optimisation. The design philosophy and the optimal design solution differ among industries. An example would be an aviation engineer trying to reduce the weight of an aeroplane or a civil engineer trying to create the most durable structure possible. During design or optimisation, attention should be paid first of all to the safety of the entire structure [23]. The aspects described above constitute an indispensable part of the structural stability loss and are taken into account in the structure design processes.

Numerous optimisation methods are in use. One of the basic methods is parametric analysis, which is based on a gradual change of successive parameters and choosing the optimal solution from the results obtained. The advantages of the method are its simplicity and suitability for simple solutions. More complicated algorithms are used and modified to describe and optimise complex problems. Their modification is often aimed at increasing the accuracy of results and shortening the optimisation time. Particularly popular are genetic algorithms (GA) and artificial neural networks (ANN).

Zenzen et al. [24] employed a two-stage approach focused on a modified transmissibility damage indicator and ANN to estimate the location and size of damage in composite structures. Khatir et al. presented a structural damage assessment technique using vibration data in order to identify the damage [25].

Typically, researchers implement a single neural network to describe a problem; however, they can be combined into groups. This leads to improving the quality of results and simplifying the resulting neural networks. The combination of artificial neural networks is commonly used in other fields of technology such as image recognition, text, speech, and translation.

In this work, we attempt to perform a numerical analysis of the optimisation of the composite laminate structure aimed to determine such a layer configuration that will display the highest resistance to stability loss. In the optimisation, the critical force and the first buckling mode were assumed as a criterium of the stability loss state. The first method determined the optimum angle of the arrangement in each laminate layer using FEM. The method has been divided into two stages to reduce the computation time. The second method employed artificial neural networks to predict critical buckling force values and create an optimisation tool. In addition, by combining the artificial neural networks into groups, the quality of the obtained results was improved and the obtained neural networks were simplified.

## 2. Purpose and Methodology of Research

Above all, the aim of the study is to determine the layout of layers of the CERP laminate providing the highest values of buckling force for the investigated composite structure. Secondly, the positive aspects of particular layups were discussed based on the given structure as well as an assessment of the selected methods (parametric studies and artificial neural network) for the structure optimisation with respect to the increasing its resistance to stability loss.

The values of critical forces for the examined composite structure were determined using the FEM linear perturbation method in the commercial programme ABAQUS^®^. This software was used to model the behaviour of composite materials and determine the eigenvalues [2,3,6,26,27,28]. The buckling loads were determined considering the base state of the structure—that is, the state of the load for which the eigenvalues are determined. In the base state, the structure may have initial loads P, stemming from previous analyses or initial conditions [2,7,29,30,31,32,33,34,35,36]. The process of determining the critical force is divided into two stages. In the first phase, the initial state of the investigated structure is determined. Further on, the eigenvalues of the structure are calculated by solving the eigenproblem equation presented below:

(1) $$\left(K_{0}+\lambda_{i}K_{\Delta }\right)v_{i}=0$$

$K_{0}$ = Stiffness matrix equal to the initial state, including the effects of the initial loads.
$K_{\Delta }$ = differential stiffness matrix for stress and load, due to incremental load Q
$\lambda_{i}$ = i-numerous eigenvalues
$v_{i}$ = i-numerous eigenvectors of the displacement (buckling form)

**Figure 1:**
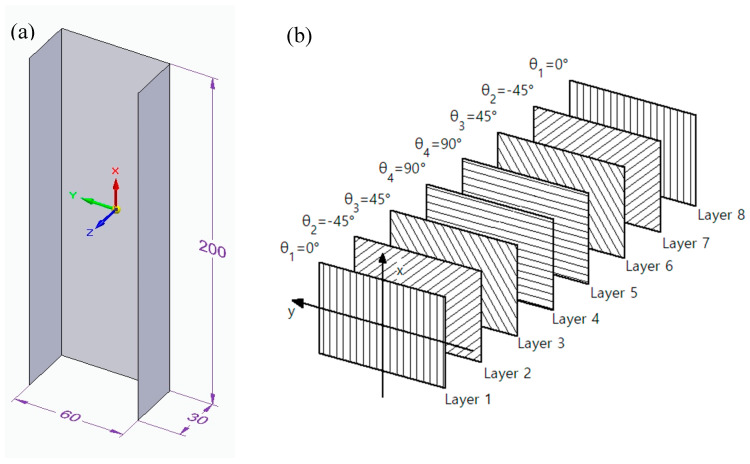
Examined composite structure: (**a**) geometrical model; (**b**) exemplary arrangement of the laminate layers, configuration [0/−45/45/90]s (own work).

**Figure 2:**
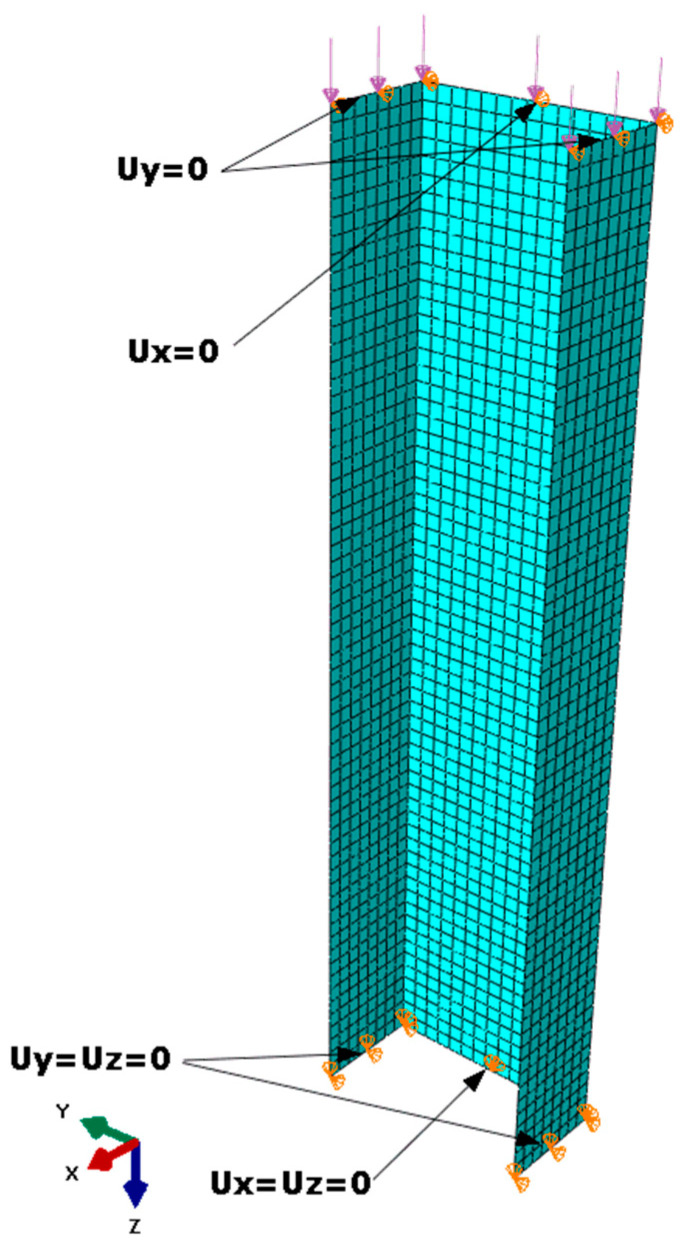
Boundary conditions from the discretised model (own work).

The research was conducted for a composite thin-walled column with a channel section-type profile made of 8 laminate layers, with the height and width of the cross-section, respectively, 60 mm and 30 mm and the profile height of 200 mm (Figure 1a). In the numerical model, it was assumed that the direction of the laminate location is parallel to the vertical axis (height) of the column and convergent with the x-axis of the local coordinate system presented in Figure 1a, on the basis of which the geometrical configuration of the layers was obtained. An exemplary configuration of layers is shown in Figure 1b. The discretisation of the geometry was conducted using an 8-node finite element surface conventional shell S8R with the shape function of the second order and reduced integration. The values of the critical forces were determined for the state of load characteristic for axial compression. The boundary conditions were formulated to ensure a simple support of the examined element, by using the displacement = 0, in the directions normal for the surfaces of each wall, in the nodes located at the edges of the end sections of the channel. Additionally, for nodes located on the edges of the lower-end section, the possibility of displacement in the channel axis directions was locked, while a uniformly distributed compressive load was applied to the edge of the upper-end section (Figure 2).

The examination was performed for symmetric layouts of the carbon–epoxy tape laminate layers. The strength properties are presented in Table 1. The thickness of a single layer was t = 0.105 mm, which is equivalent to the thickness of a single layer of the material of which the elements described further in this study are composed.

**Figure 3:**
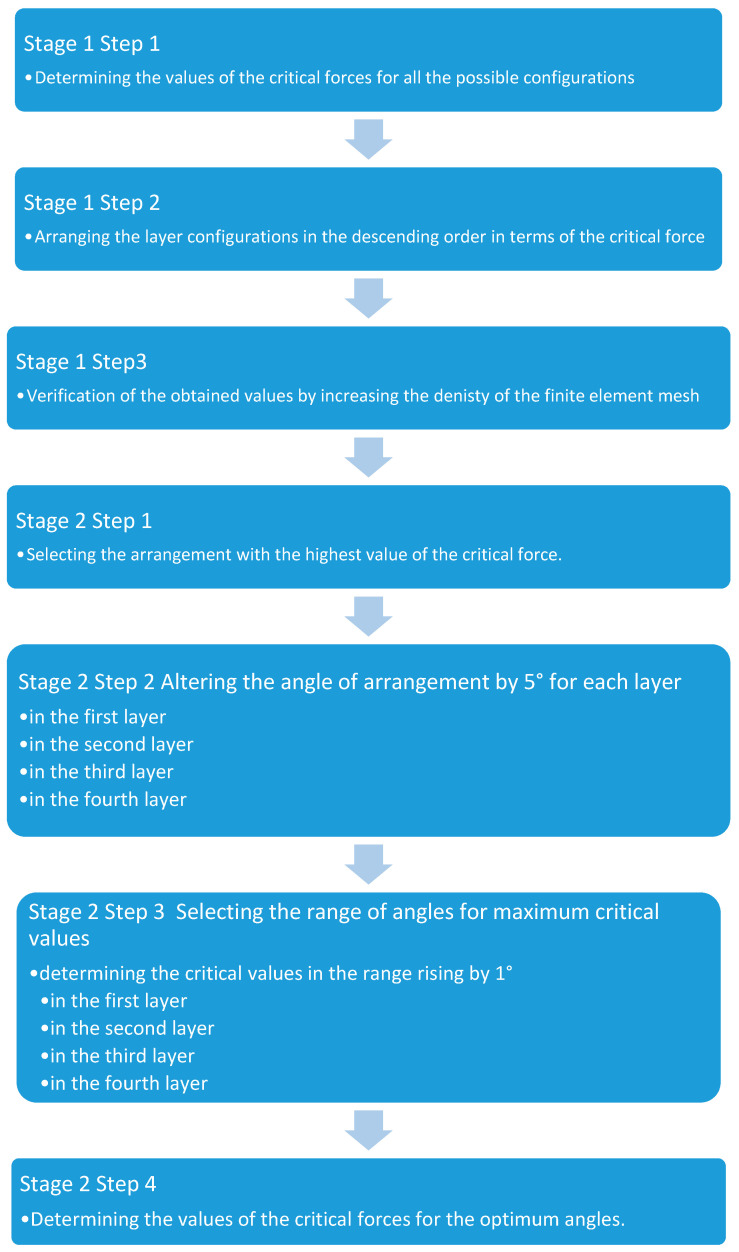
An algorithm for the determination of layer configuration for the highest critical force.

The determination of the layer configuration with the maximum value of the critical force was divided into two stages (Figure 3). In the first stage, the values of critical forces for selected, and symmetric configurations of laminate layers for a 200-mm-long column were determined using parametric examination [3,5,37,38,39,40,41,42,43]. Moreover, similar tests were conducted for profiles with lengths equal to 400, 600, 800, and 1000 mm, which enabled comparing the layer configuration at different lengths of the examined profile. During the determination of the configurations, it was assumed that a single layer can be placed in four typical directions, i.e., the angle of layer placement θ∈{0°, 90°, 45°, −45°}. Limiting the possible directions of layer placement and laminate symmetry allows for 256 combinations. It is to be noted that due to the geometric symmetry of load and the geometry, the layouts in which the directions 45° and −45° occur subsequently, e.g., configuration [−45/45/0/90]s, is the symmetry of the configuration [45/−45/0/90]s. The predicted values of the critical forces for the possible layouts of the laminate layers are compared and presented in a respective table. Subsequently, the values were arranged in descending order of critical force, creating a ranking of layer configurations. At the end of this stage, the obtained results were verified by increasing the density of the finite element mesh by two or four times for the configurations from the top of the ranking [44]. At the second stage, the arrangement angle θ (Figure 1b) in each layer was changed in the range 0° to 90° at 5° steps and beginning from the first, outer layer and ending with the fourth layer, located in the centre (Figure 3). Next, for the ranges with the highest value of the critical force obtained from the charts, calculations were carried out for the increase of one degree in each layer to determine the optimum angle of the layer arrangement. This stage determined the value of the critical force for the optimum laminate layer angles, creating the optimum configuration in terms of the value of the critical force (Figure 3).

## 3. Results from the First Optimisation Method

### 3.1. Determining the Arrangement of Layers with a Maximum Value of the Critical Force

Table 2 presents 20 configurations of layers with maximum values of the critical force for the 200 mm-high column. The arrangement with the highest critical force values was [−45/45/45/−45]s, placing the first and second in the ranking. The arrangement [−45/45/−45/45]s, frequently used in the production of laminate elements, placed 10th and 11th, which is indicative of its low resistance to stability loss.

The critical force for the first arrangement is higher than in the most frequently applied arrangement by 5%. Furthermore, the arrangements with the highest critical force values have the first two layers in the arrangement [45/−45/…]s.

As in the 200 mm column, the values of the critical forces were determined and arranged for the remaining lengths of the analysed profile—that is, 400, 600, 800, and 1000 mm.

**Figure 4:**
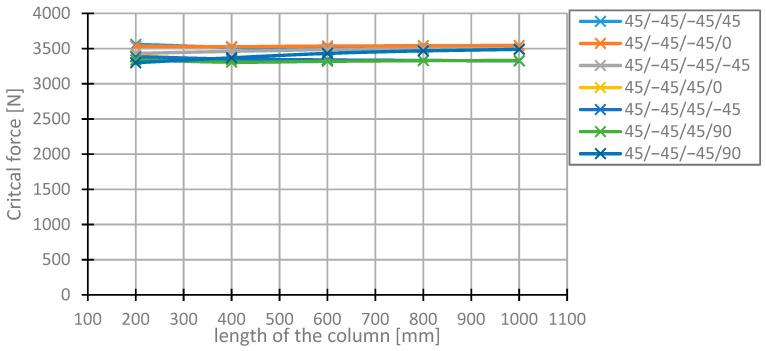
Changes in the value of the critical force for different arrangements of the laminate layers and different lengths of the column.

**Figure 5:**
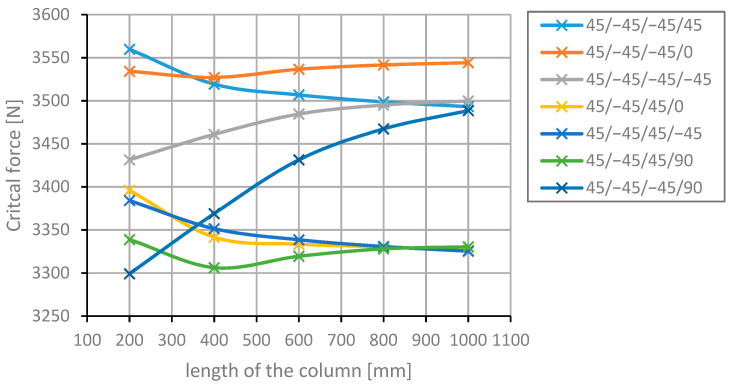
Changes in the value of the critical force for different arrangements of the laminate layers at different lengths of the column.

Figure 4 and Figure 5 present the progress of changes to the critical forces for the arrangements with the highest values of the forces in question, depending on the length of the column. The level of these forces remains nearly constant, which equals the local stability loss of thin-walled rods [45,46].

The forces remain nearly unchanged, which, according to the theory [45,46], is equivalent to the local stability loss of thin-walled rods. Upon closer inspection, that occurs due to the change in the range of values on the abscissa in Figure 5.

It can be observed that the ranking of the arrangements with the highest values of the critical force changes along with the length of the column. A good example of this phenomenon is the arrangement [45/−45/−45/90]s, which ascends from the 7th place at 20 mm to the 4th place at 400 mm. The value of the critical force of this arrangement increases rapidly. At a column length of 1000 mm, it does not differ significantly from the arrangements ranking second and third. The value of the critical force for this arrangement rose by 6% in terms of critical force at the length equal to 200 mm. Critical forces in the arrangements [45/−45/−45/45]s and [45/−45/45/−45]s, differing in the direction of placement of the third layer, decrease along with the increase of the length of the column in a similar manner. The difference in the critical force values for the columns with the lengths of 200 and 1000 mm is 5%. The layer arrangement [45/−45/−45/0] for the section lengths from 400 to 1000 mm is the arrangement with the maximum critical force. In the case of the longest column, the critical force value is higher by 6.5% in relation to the most typical layer arrangement [45/−45/45/−45]s.

### 3.2. Verification of the FEM Calculations

**Figure 6:**
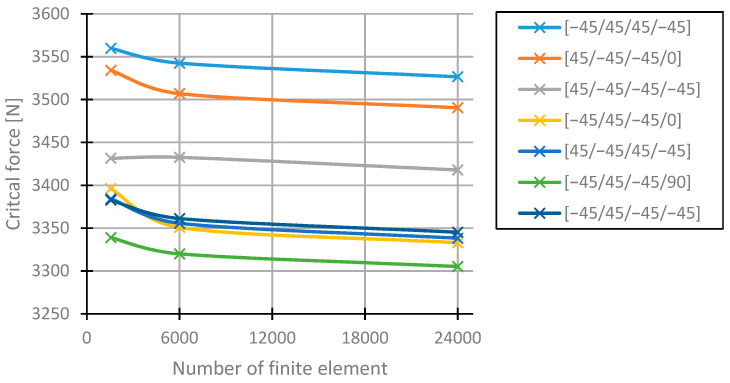
Values of the critical forces for different layer arrangements for different levels of finite element methods (FEM) mesh densification.

In order to verify the obtained results using the FEM method, additional calculations were conducted. Given that the results are approximated and their accuracy increases along with the number of finite elements, the mesh density was increased for seven arrangements with the highest values of critical forces for the column with the length of 200 mm. The basic model was discretised using 1550 finite elements; after increasing the mesh density, the number of finite elements was equal to 6000 and 24,000, which corresponds to increasing the mesh density two and four times. The obtained values of critical forces depending on the mesh density are presented in Figure 6. These values decreased for all the considered arrangements. During the first increase in the mesh density, the arrangements [45/−45/45/−45]s and [−45/45/−45/90]s changed their positions in the ranking and remained at the second increase of mesh density. The differences between these and the arrangement [−45/45/−45/0]s are insignificant. The obtained values of the critical forces are not the highest; therefore, these arrangements take ex aequo fourth place. The differences in the values of critical forces between each layer arrangements remain the same.

Based on the typical arrangement [45/−45/45/−45]s, since the value of the critical force does decrease in this case and the differences remain unchanged, the percentage increase of the critical force rises. Due to the changes in the ranking, the obtained results ought to be verified by increasing the density of the FEM mesh.

The second stage was divided into four steps, in accordance with the algorithm (Figure 3). The first step comprised of selecting a layer configuration with the highest values of critical force from stage one, mainly [45/−45/−45/45]s. In the second step, the angle θ (Figure 1b) was altered for the laminate layers 1 to 4 (outer to central layer) from 0° to 90°, at 5° steps. Next, the ranges of angles with the highest critical force were established. For those ranges, the calculations with the increase of the angle by one degree were conducted.

**Figure 7:**
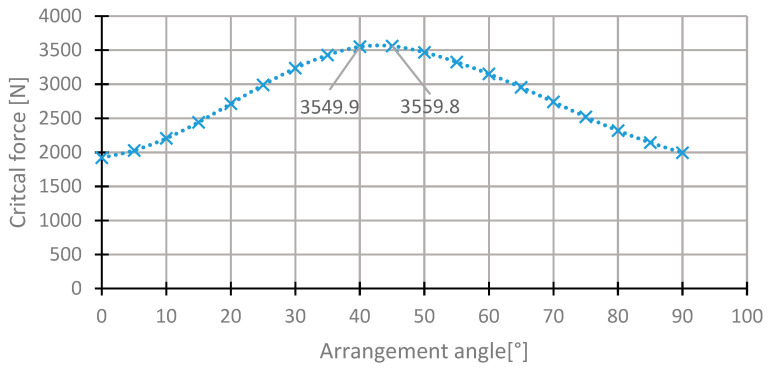
The critical force depending on the angle in the first layer of the [45/−45/−45/45] arrangement.

**Figure 8:**
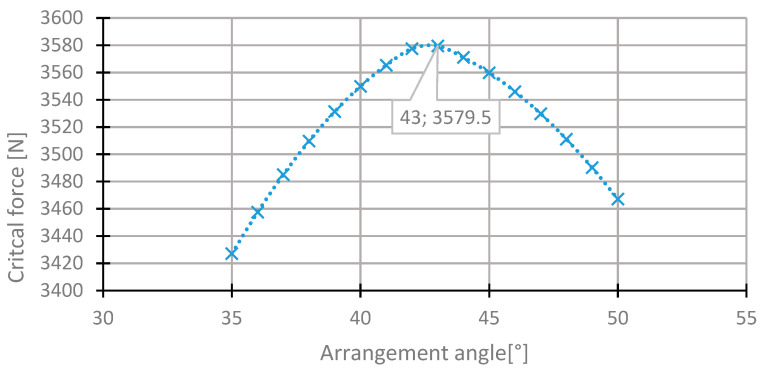
The critical force depending on the angle in the first layer of the laminate [45/−45/−45/45] for the change of the arrangement in the range 35°–50°.

For the first layer, the highest value of the critical force was in the range of 35°–50° (Figure 7). The value of the critical force changes from 2000 to 3560 N. The highest value was obtained for the initial direction, 45°. The angle of maximum critical force was 43° (Figure 8). For this arrangement, the critical force is higher by 0.53% than in the initial arrangement.

**Figure 9:**
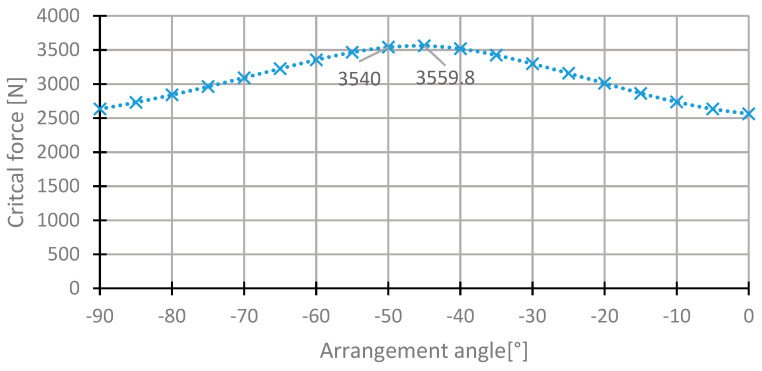
The critical force depending on the angle in the second laminate layer of the [45/−45/−45/45] arrangement for the change of the arrangement angle in the range from −90° to 0°.

**Figure 10:**
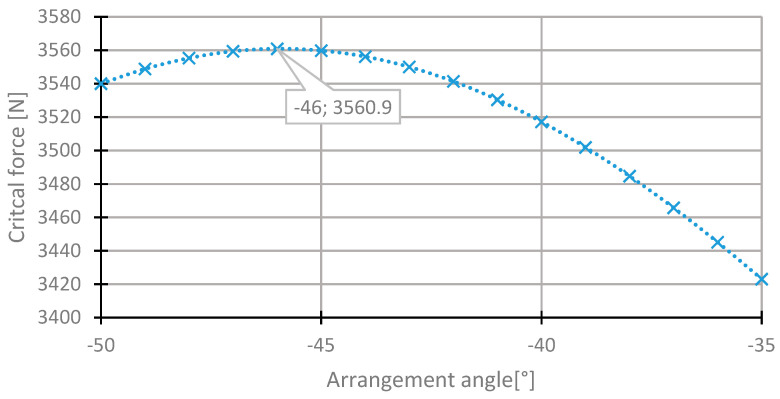
The critical force depending on the angle in the second laminate layer of the [4 5/−45/−45/45] arrangement for the change of the arrangement angle in the range from −50° to 35°.

In the second layer, the change in the angle θ_2_ occurred in the range −90° to 0° in order to keep the direction of the arrangement from the initial sequence of the layers. For this layer, the angle with the maximum critical force was observed in the range from −50° to −35° (Figure 9), similarly to the first layer. The highest value of the critical force was observed for the angle −46° (Figure 10). The increase in the critical force for this change is insignificant, approximately 0.005%.

**Figure 11:**
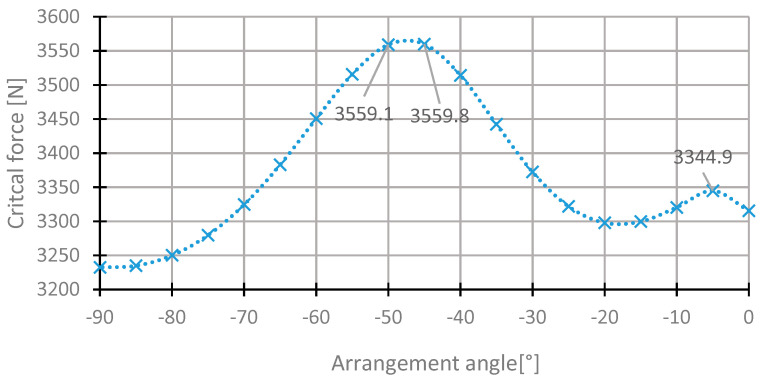
The critical force depending on the angle in the third laminate layer of the [45/−45/−45/45] arrangement for the change of the arrangement angle in the range from −90° to 0°.

**Figure 12:**
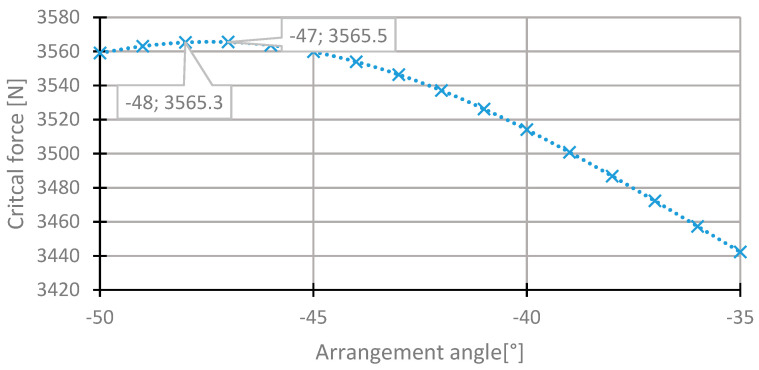
The critical force depending on the angle in the third laminate layer of the [45/−45/−45/45] arrangement for the change of the arrangement angle in the range from −50° to 35°.

The range of the angle θ_3_ change is similar to the case of the second layer. The highest value of the critical force was observed for the angle of −47°. The critical force is higher than in the case of the basic configuration by 0.16%. The distributions of the critical force depending on the angle of arrangement in the third layer are presented in Figure 11 and Figure 12.

**Figure 13:**
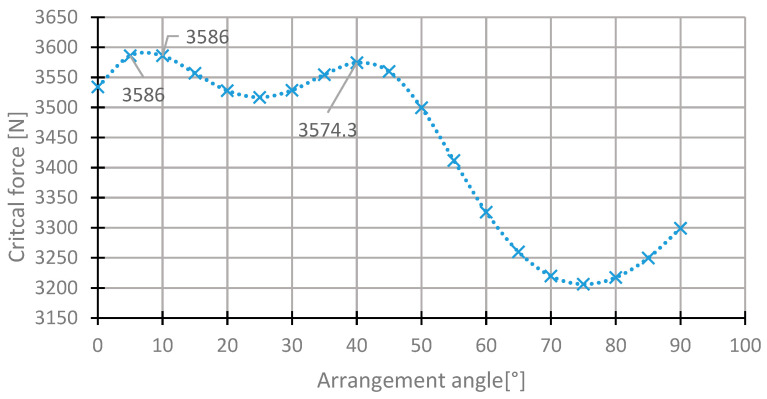
The critical force depending on the angle in the fourth laminate layer of the [45/−45/−45/45] arrangement for the change of the arrangement angle in the range from 0° to 90°.

**Figure 14:**
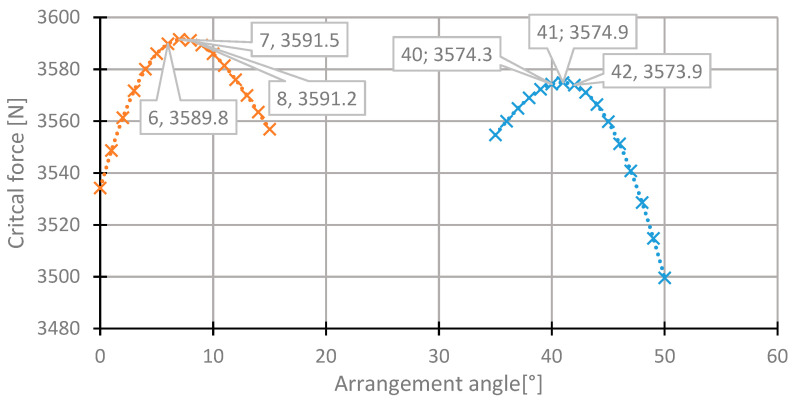
The critical force depending on the angle in the fourth laminate layer of the [45/−45/−45/45] arrangement for the change of the arrangement angle in the ranges 0°–15° and 35°–50°.

In the fourth central laminate layer, the critical forces are the highest in the two ranges. The first one is 0° to 15°, and the second one is 35° to 50° (Figure 13). For this reason, both ranges were subjected to a more thorough analysis (Figure 14). The maximum critical force was obtained for the angle of 7°. The critical force for this angle was higher than in the case of the initial state by 0.89%. The angle from the second range was 42°.

Eventually, the calculations were conducted for the arrangement with the angles with maximum critical forces, i.e., [43/−46/−47/7]s. The value of the critical force for this arrangement was the highest among the results for a 200 mm column, and it was 0.98% higher than in the case of the initial arrangement. Referring to the frequently used arrangement [45/−45/45/−45]s, the critical force rose by 6.38%.

## 4. Optimisation Using Artificial Neural Networks

The data obtained using the method described in Section 2 served as a database for designing the artificial neural networks. Subsequently, the ANNs were used to solve the optimisation problem: searching for a layer arrangement with the highest critical force. The artificial neural networks were modelled using STATISTICA^®^ software. The output quantity variable was the value of the critical buckling force, whereas the input quantity variables were the angles of each laminate layer θ as well as the height of the column L. The sampling method was a random sample, in which the samples used for teaching ANN (teaching set), testing (testing set), checking the learning effectiveness, and validation (validation set) were selected in a random manner. Validation determined the predictive accuracy of the tests that were not used for teaching or testing the network.

The teaching set constituted 70% of all the samples, whereas the testing and validation sets constituted 15% each at the initial value of the random number generator equal to 1000. In order to create the network, a two-layer multilayer perceptron (MLP) was used, being the most commonly used type of network. This iterative learning type of network considers the following transfer functions: linear, logistic, hyperbolic tangent, exponential, sine, hidden, and output neurons. The initial weight values were determined by selecting the value of the random number generator by 1000. In the teaching, the BFGS (Broyden–Fletcher–Goldfarb–Shanno) algorithm was used. The error function used for the assessment of the network quality was the sum of squares. The obtained networks are presented in Table 3.

(2) $$E_{sos}=\overset{N}{\underset{i=1}{\sum }}{\left(y_{i}-t_{i}\right)}^{2}$$

where: *N*—number of cases, *y_i_* —prediction of networks, *t_i_*—real value (from the data), *i*—case number.

The network quality is expressed by the correlation coefficients between the output variable and the value predicted by the network. For all of the networks, the quality value exceeded 0.99. Teaching errors for each group are in the range from 900 to 1500. Each obtained network contains a hyperbolic tangent in the hidden layer, whereas in the output layer, the first and the last network uses sine as the transfer function. The second and the third network uses the linear function, whereas in the fourth one, the hyperbolic tangent is used as well. The number of neurons in the hidden layer ranges from 25 to 30, which is the maximum allowed number of neurons.

Table 4 presents the results from the Global Sensitivity Analysis of the obtained networks. The values in the table present the quotient determining how the error of the network will increase after removing the variable from the input data. For each network, the least significant variable is the angle of laminate arrangement in the central layer θ_4_ and the length of the column. The most important factor according to the networks 1, 2, 4, and 5 is the ply angle θ_1_ in the first layer. Network 3 was found to be the most sensitive to the ply angle θ_2_ in the second layer. Based on the obtained sensitivity analysis, it can be stated that the laminate ply angle is the more important the more remote it is from the surface of the geometric symmetry. Similar conclusions can be drawn based on the charts presented in Figure 8, Figure 10, Figure 12 and Figure 14, and more specifically the range of the change to the value of the critical buckling force.

### 4.1. Numerical and Experimental Prediction of Critical Force Values

**Figure 15:**
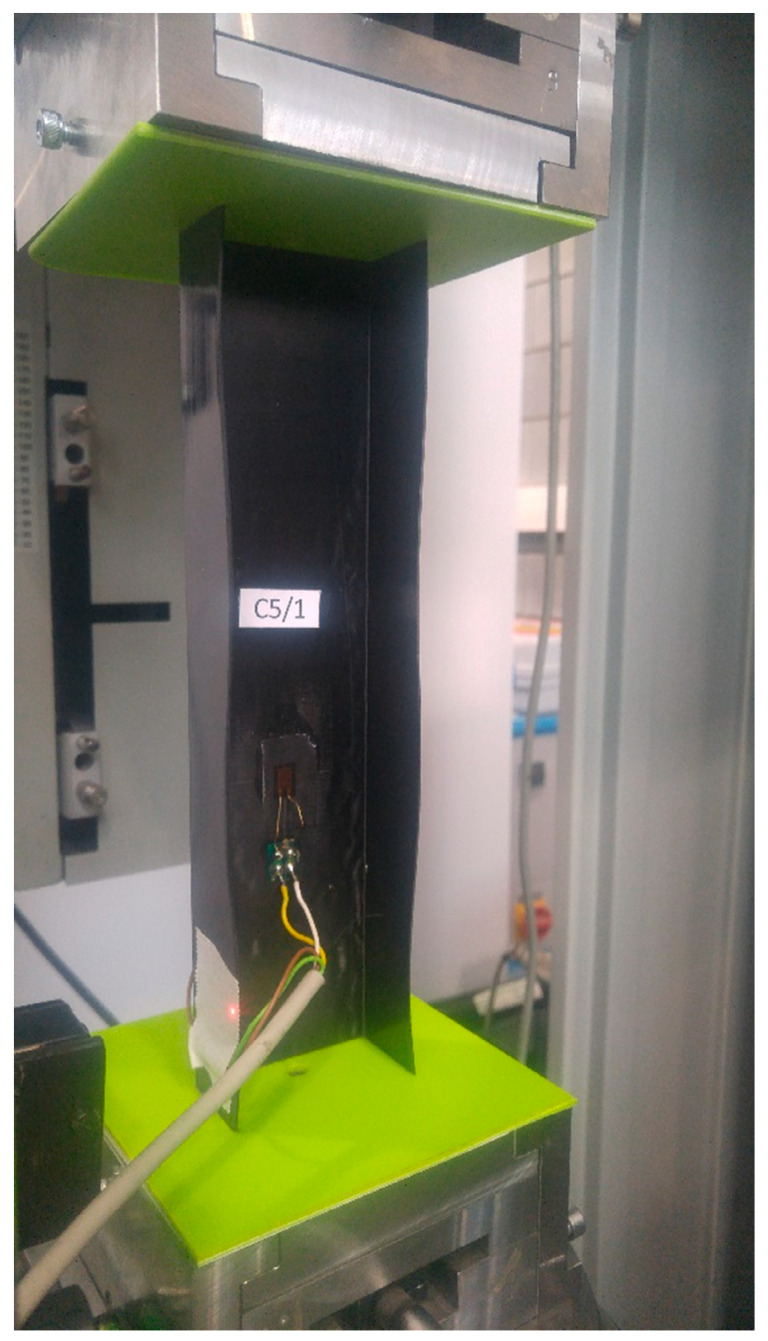
Axial compression test of the 250 mm column and layer configuration [90/0/90/0]s.

The obtained networks were used to predict the value of the critical bucking force for the following layer configurations: [0/45/−45/90]s, [90/−45/45/0]s, [45/−45/90/0]s, [90/0/90/0], and the length of the column equal to L = 250 mm. The selected configurations were considered in the teaching process or the validation of the neural networks. The single new aspect was the height of the column. The obtained values are summarised in Table 5 along with the values obtained using the finite element method and the experimental methods Pw^2^ and Koiter. The experimental testing (Figure 15) was thoroughly described in other works [47,48]. The predicted values of the critical forces are fairly similar to the values obtained using other methods. These values contain certain errors, differences to be more precise, albeit insignificant ones.

### 4.2. Optimisation Using Neural Networks

The models of neural networks were used for optimising of the configuration of the laminate layers. A configuration with the possibly highest value of the critical force is sought. In order to check the usefulness of the neural networks in solving the optimisation problem, the optimisation process was conducted using the simplex technique and mesh. The process was conducted separately for each network as well as after grouping.

#### 4.2.1. Optimisation Using the Simplex Algorithm

**Figure 16:**
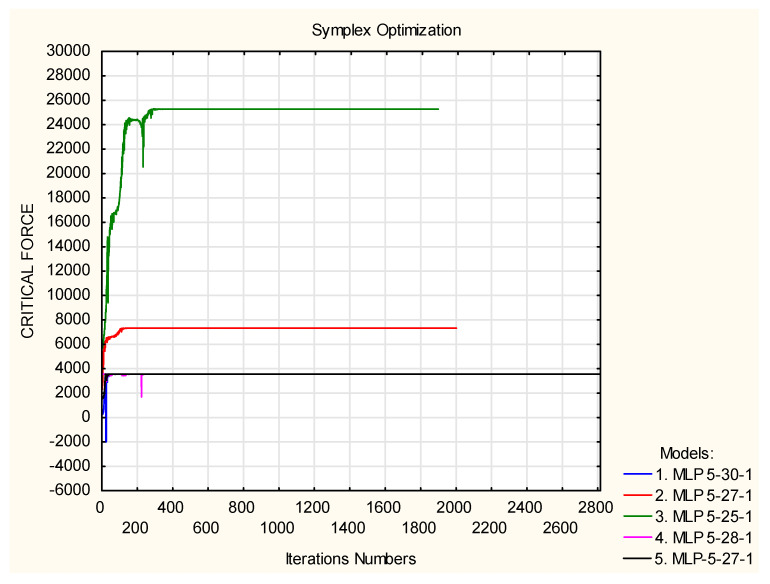
Values of the critical forces [N] in iterations.

In the first attempt, the simplex algorithm (iterative) was used. The maximum iteration number was 10,000 and the alloying criterion 0.0001, which means that the algorithm is stopped should the differences between subsequent iterations be smaller than the value of the alloy. The predicted critical values for different networks are shown in Figure 16. The predictions for the second and third networks are significantly higher than the rest; for this reason, it was decided to exclude those networks from the optimisation processes. The values of the ply angle configurations and the critical values of the buckling forces are presented in Table 6. The values of the critical forces obtained from the prediction of the neural networks differ significantly from the ones obtained using FEM. The applied algorithm found optimisation solutions outside of the range of values of the variable used in the teaching process, i.e., [−45°,90°]; therefore, the obtained results are erroneous.

Another optimisation was conducted only for the networks that reached the predicted value of the critical force in the previous analysis. These are the networks 1, 4, and 5. In the case of the MLP 5-30-1 network, the optimal layer configuration is similar to the one obtained in the previous analysis. The optimal configuration of angles for the remaining networks remains unchanged. The values of the obtained angles do not exceed the range of angles [−45°, 90°] used in the teaching process. However, the verification of the obtained angles was conducted using FEM, and in this case, the significance in the angle values can be observed. The obtained results are presented in Table 7.

Another optimisation was conducted by grouping three networks with similar values of the critical force. This operation allowed for a joint prediction of the dependent variable for the set of values of the dependent variables [49]. The group of networks exhibited better performance than the best network in the group. Despite the fact that the angle values in the optimum configuration insignificantly exceed the range of the input data, the value of the predicted critical force is higher by only 3% in relation to the value obtained by FEM (Table 8).

#### 4.2.2. Optimisation Using the Mesh Method

The last optimisation was performed using the mesh method. It is more fastidious in terms of calculation than the simplex method, due to a higher number of calculations. The optimisation was performed by creating two groups of networks. The first group consisted of all five networks, whereas the second one consisted of three networks. In the case of the first group, the predicted critical force was higher than the one obtained with FEM by 23%, whereas in the second group, it was 10% higher (Table 9).

## 5. Discussion

In the first part of this study, an attempt at using parametric testing to determine the arrangement of composite layers with the highest critical force was made. The approach presented in this work is an optimisation solution aiming to determine such a laminate arrangement for which the optimised structure would be the most resistant to stability loss. The optimisation was achieved by assuming the invariability of the geometry of the optimised structure, the state of load, and the mechanical properties of the composite material. In the first stage of the algorithm (Figure 3), the best laminate configuration [−45/45/45/−45]s was determined, for which the critical force is higher than for the cross arrangement [45/−45/45/−45]s by 5%. By switching the layer directions in the two first layers, it is possible to increase the resistance to stability loss by 5% at a constant mass. It is also to be noted that switching directions of the layer arrangement does not require any technological operations; therefore it is easy to perform. The verification of the obtained results, consisting of increasing the density of the finite element mesh, showed that the values of the critical forces decrease along with the increase in the number of finite elements. However, the difference remains unchanged. Therefore, the percentage benefit from using the optimal configuration of layers increases.

In the second stage, the layup angle was changed in each layer, based on the configuration selected in the first stage, [−45/45/45/−45]s. The percentage benefit from applying the selected layers does not exceed 1% at this point. The greatest increase of the critical force was observed in the change to the angle in the fourth, central layer, 0.89% for the angle of 7°. In the first layer, changing the angle from 45° to 43° produced a critical force higher by 0.53%. Changes to the angles in the second and third layer increased the critical force by, respectively, 0.005% and 0.16%. The critical force of the configuration of layers [43/−46/−47/7]s is higher by 0.98% in relation to the arrangement from the first stage and by 6.38% in relation to the cross arrangement [45/−45/45/−45]s. Considering the manufacturing process, for the optimised structure, the layer arrangement in the second stage may prove inefficient. However, the benefits from applying the second stage may be greater for other composite structures, e.g., laminated shells and plates or the same structure but with other cross-section dimensions.

An analysis of the layer arrangement for different heights of the column showed that depending on the height of the column, the layer arrangements with the highest critical force have different angle configurations [29]. Thus, the optimum angle configurations are sensitive to the geometrical parameters of the optimised structure.

In the second part of the study, artificial neural networks were used in the optimisation process. The optimisation methods are simplex and mesh. The predicted values of critical buckling force and the configuration of the laminate layers, using the simplex method, separately for each network, differed significantly from the values of force obtained using FEM. The highest convergence was obtained for the simplex method, upon joining the networks in a group. The difference in the critical forces was 3%. The optimisation using the mesh method, depending on the numbers of networks in a group, produced the differences of 23% and 10%. The values of the critical buckling forces of the optimum configurations did not exceed the values obtained in the first part of the study.

## 6. Conclusions

The study presents an optimisation approach to the thin-walled composite structures in terms of stability loss. The parametric testing showed that depending on the geometric parameters, the layer configurations with the highest critical force vary. The proposed optimisation methods determined the configuration of angles for which the maximum critical force is the highest. Using artificial neural networks did not influence the determination of the optimum laminate configuration. Increasing the acceptable number of neurons in the hidden layer and applying other methods in the teaching process may increase the network accuracy. The best optimisation results were obtained by combining the networks in groups. The sensitivity analysis confirmed that the directions of arrangement in the outer layers have the most significant influence on the critical buckling force. It is a utilitarian conclusion. The significance of the arrangement is greater the more remote the layer is from the surface of the geometrical symmetry. Using the artificial neural networks for the optimisation of the composite structure requires experience and experimental verification. The results obtained using this method are affected by errors dependent on the quality of the networks and the teaching sets in terms of qualitative and quantitative variables.

## Figures and Tables

**Table 1:** Strength properties of the lamina.

E_1_ [GPa]	E_2_ [GPa]	ν_12_ [–]	G_12_ = G_13_ = G_23_ [GPa]
143,527	5.826	0.36	3.845

**Table 2:** Arrangement of the layers with the highest values of the critical force.

No.	θ_1_	θ_2_	θ_3_	θ_4_	Critical Force [N]
1	−45	45	45	−45	3559.8
2	45	−45	−45	45	3559.8
3	45	−45	−45	0	3534.2
4	−45	45	45	0	3534.2
5	45	−45	−45	−45	3431.5
6	−45	45	45	45	3431.5
7	−45	45	−45	0	3396
8	45	−45	45	0	3396
9	45	−45	45	−45	3384.1
10	−45	45	−45	45	3384.1
11	−45	45	−45	−45	3382.6
12	45	−45	45	45	3382.6
13	−45	45	−45	90	3338.9
14	45	−45	45	90	3338.9
15	−45	45	0	−45	3315.3
16	45	−45	0	45	3315.3
17	45	−45	0	90	3312.8
18	−45	45	0	90	3312.8
19	45	−45	0	−45	3311.1
20	−45	45	0	45	3311.1

**Table 3:** Comparison of the best neural networks.

	Network Name	Quality (Teaching)	Quality (Testing)	Quality (Validation)	Error (Teaching)	Error (Testing)	Error (Validation)	Teaching Algorithm	Error Function	Transfer (Hidden)	Transfer (Output)
1	MLP 5-30-1	0.9974	0.9969	0.9969	1057.2	1253.9	1332.2	BFGS 316	SOS	Tanh	Sine
2	MLP 5-27-1	0.9977	0.9971	0.9974	924.18	1185.8	1131.5	BFGS 651	SOS	Tanh	Linear
3	MLP 5-25-1	0.9970	0.9969	0.9967	1210.1	1254.3	1421.4	BFGS 350	SOS	Tanh	Linear
4	MLP 5-28-1	0.9973	0.9969	0.9967	1071.3	1298.2	1413.3	BFGS 409	SOS	Tanh	Tanh
5	MLP 5-27-1	0.9968	0.9971	0.9969	1297.4	1208.2	1352.2	BFGS 482	SOS	Tanh	Sine

**Table 4:** Results from the global sensitivity analysis of the obtained neural networks.

Networks	θ_1_	θ_2_	θ_3_	θ_4_	L
1.MLP 5-30-1	268.4075	190.4372	75.00891	2.705788	2.306382
2.MLP 5-27-1	360.4077	254.5779	81.06350	6.753971	2.433762
3.MLP 5-25-1	215.1952	357.2611	43.63368	5.776083	2.269453
4.MLP 5-28-1	669.0061	299.1550	48.76469	2.647628	2.235855
5.MLP 5-27-1	341.3829	276.8474	45.49597	2.452796	2.267050
Mean	370.8799	275.6557	58.79335	4.067253	2.302500

**Table 5:** The values of the critical buckling forces predicted by the artificial neural networks with the values obtained using the finite element and experimental methods.

Configuration	Values of Critical Forces							
	Neural Networks					FEM	Stand Experiment	
	1	2	3	4	5		Pw2	Koiter
[0/45/−45/90]s	2186.2	2218.1	2235.7	2169.0	2218.0	217,004	21,592	21,241
[90/−45/45/0]s	2189.0	2174.5	2218.2	2205.2	2181.6	21,817	21,672	21,249
[45/−45/90/0]s	3278.9	3224.0	3295.3	3269.8	3242.7	32,599	31,968	31,968
[90/0/90/0]s	1598.8	1602.9	1649.2	1578.9	1520.	15,195	15,787	15,783

**Table 6:** The values of the optimum angle configurations determined for each neural network as well as the values of the predicted critical forces and obtained using FEM.

Network Number	θ_1_	θ_2_	θ_3_	θ_4_	Predicted Critical Force	FEM Critical Force
1. MLP 5-30-1	68.4	−45.4	52.4	15.1	3559.62	2603.8
2. MLP 5-27-1	96.6	−124.9	12.0	17.0	7323.32	1900
3. MLP 5-25-1	170.5	−1217.8	329.5	−45.0	25,269.05	2245.9
4. MLP 5-28-1	−45.0	−45.00	−71.5	23.3	3557.35	1327.6
5. MLP 5-27-1	−105.9	−10.75	−44.1	−44.3	3559.69	2066.2

**Table 7:** The values of the optimum angle configurations determined for each neural network as well as the values of the predicted critical forces and obtained using FEM.

Network Number	θ_1_	θ_2_	θ_3_	θ_4_	Predicted Critical Force	FEM Critical Force
1. MLP 5-30-1	68.46772	−45.3968	52.41154	15.10200	3559.617	2603.8
4. MLP 5-28-1	90.00000	−98.2066	31.03769	63.67580	3521.089	1184.6
5. MLP 5-27-1	65.77956	−47.5496	39.75084	32.79484	3559.590	26,690

**Table 8:** The values of the optimum angle configurations, determined for group neural network as well as the values of the predicted critical forces and obtained using FEM.

	θ_1_	θ_2_	θ_3_	θ_4_	Predicted Critical Force	FEM Critical Force
Network group	41.3	−50.8	−55.6	47.0	3548.3	3415.4

**Table 9:** The values of the optimum angle configurations determined for group neural network as well as the values of the predicted critical forces and obtained using FEM.

	θ_1_	θ_2_	θ_3_	θ_4_	Predicted Critical Force	FEM Critical Force
Network group 1	−45	30	65	15	3763.7	3088.8
Network group 2	52	−45	−45	−45	3514.2	3178.8
